# Barriers to and facilitators of employment of persons with disabilities in low- and middle-income countries: A scoping review

**DOI:** 10.4102/ajod.v10i0.833

**Published:** 2021-06-22

**Authors:** Refilwe E. Morwane, Shakila Dada, Juan Bornman

**Affiliations:** 1 Centre for Augmentative and Alternative Communication, Faculty of Humanities, University of Pretoria, Pretoria, South Africa

**Keywords:** barriers, disability, employment, facilitators, International Classification of Functioning Disability and Health (ICF) framework, low- and middle-income countries (LMICs), societal participation

## Abstract

**Background:**

Unemployment rates for persons with disabilities in low- and middle-income countries (LMICs) are high. This is despite the call to action by the United Nations Convention on the Rights of Persons with Disabilities and Sustainable Development Goals aimed at improving the economic well-being of the marginalised. To improve the employment outcomes of persons with disabilities in these countries, factors that facilitate and hinder employment should be explored.

**Objectives:**

This study explored barriers to and facilitators of employment for persons with disabilities in LMICs through a scoping review.

**Methods:**

A search strategy included a systematic search of nine databases using specific keywords. The International Classification of Functioning, Disability and Health (ICF) framework was used as a conceptual framework and barriers and facilitators were reported according to the domains of the ICF. Articles published between 2008 and 2020 were reviewed using a predefined criteria.

**Results:**

Thirty-two studies were identified in the review. Factors were identified in all domains of the ICF: (1) body function and body structure (12; 39%); (2) activities and participation (13; 42%); (3) personal factors (23; 74%); (4) environmental factors (27; 84%).

**Conclusion:**

Factors that hinder and facilitate the participation of persons with disabilities in LMICs were mainly found in the environment, with personal factors also influencing participation. The presence of negative attitudes and lack of services mainly in health and transport were major factors within the environment whilst personals factors included the lack of educational qualifications and skills. These results indicate the importance of consideration of contextual factors when developing intervention strategies aimed at facilitating the employment of persons with disabilities in LMICs.

## Introduction

People with disabilities constitute approximately 15% of the world’s population, a rising figure compared to the 10% prevalence rate estimated in the 1970s (WHO [World Health Organization] and World Bank 2011). A significant proportion of these individuals live in low- and middle-income countries (LMICs) where unemployment rates for persons with disabilities can be as high as 60% – 90% (United Nations Flagship Report 2018). Indeed, both the prevalence and unemployment rate of persons with disabilities vary amongst countries and are significantly influenced by the political, social and economic status of that country (Jenkins et al. 2011).

The World Report on Disability (WHO and World Bank 2011) describes barriers faced by persons with disabilities which result in exclusion and restrictions for participation in various live activities, such as the presence of negative attitudes, lack of delivery and provision of services, lack of accessibility, inadequate funding and lack of consultation of persons with disabilities themselves. Mitra, Posarac and Vick (2013) gave a snapshot of the economic well-being of persons with disabilities in 15 LMICs. The results of the study indicated that persons with disabilities presented with low education, low participation in the workforce and lived in abject poverty. These results are similar to previous studies that have reported a link between disability and poverty (Banks, Kuper & Polack 2017). In most instances, the source of income emanates from social security benefits or grants. It is therefore not surprising that persons with disabilities are the most economically disadvantaged group in society, particularly those in LMICs (Mitra et al. 2013). Employment is considered a mode of societal participation and therefore extends far beyond economic sustainability as it facilitates inclusion and participation in everyday life activities (Hästbacka, Nygård & Nyqvist 2016). Given the consequences of non-participation in the economic environment, unemployment of persons with disabilities then becomes a violation of human rights.

With the world report on disability (WHO and World Bank 2011) recommending practical solutions to the current barriers faced by persons with disabilities, some governments in LMICs heeded the call to action and responded with the drafting of policies and programmes that promote the participation of persons with disabilities, particularly in areas related to education, health and employment (Cobley 2013). Despite these initiatives, persons with disabilities continue to be side-lined and face barriers in accessing health services, education and employment opportunities (Mitra & Sambamoorthi 2014).

In order to propose strategies that promote and improve the employment outcomes of persons with disabilities in LMICs, an understanding of factors that hinder and facilitate their employment is required. Currently, evidence regarding this is based on literature from high-income countries (HICs) (Harmuth et al. 2018; Khayatzadeh-Mahani et al. 2019; Vornholt et al. 2018). According to the social model of disability, disability is a result of barriers that exist in the social, economic and attitudinal environment and not because of the impairment in health conditions (Oliver 1990). Therefore, an individual is disabled because of barriers that exist in that specific environment which is context-bound.

Comparatively, barriers identified in LMICs may differ from HICs mainly because of the availability of resources and sustainable services (WHO and World Bank 2011). In most LMICs, the lack of availability of quality prevalence data because of inconsistent use of the definition of disability, amongst others, results in data that are incomparable internationally (Schneider & Nkoli 2011). Therefore, data cannot be easily transferred from one context to the other. There are limited studies that have systematically reported on what hinders and facilitates the employment of persons with disabilities in LMICs (Ebuenyi et al. 2018; Mizunoya & Mitra 2013; Tripney et al. 2019; Visagie et al. 2017).

Recently, a scoping review by Ebuenyi et al. (2018) reported on barriers to and facilitators of employment of persons with psychiatric disabilities specifically in the African context. Poor health, social stigma, discrimination, negative attitudes from employers and lack of social support from the government were identified as the main barriers for this population in accessing employment. Conversely, facilitators included personal factors such as positive self-esteem, other forms of employment such as supported and competitive employment and reasonable accommodation in the workplace. Results further highlighted existing challenges in the development of legislation and the implementation of policies and guidelines that support the participation of persons with disabilities in the labour market in Africa. Only eight studies were included in the review (1990–2018) highlighting the paucity of research in the field of disability and employment in LMICs. In the review by Tripney et al. (2019) on the effectiveness of various intervention programs in facilitating participation in the labour market of adults with intellectual and physical disabilities from LMICs, participants reported ill-health and poor well-being, attitudinal barriers, inaccessible working environments and the lack of education and job-related skills as employment barriers post-intervention.

Although the two reviews provide some understanding of the barriers to and facilitators of employment, Ebuenyi et al. (2018) focused on psychiatric disabilities whilst Tripney et al. (2019) reported on outcomes of employment intervention programmes. The aim of this review is, therefore, to explore the complexity of participation of persons with various disabilities in LMICs by using a framework that understands the complexity of factors that hinder the employment of persons with disabilities. Studies in LMICs suggest that environmental factors are important considerations in understanding barriers or facilitators to employment for persons with disabilities (Mizunoya, Yamasaki & Mitra 2016).

The International Classification of Functioning, Disability and Health (ICF) framework (WHO 2001) describes disability as occurring at three levels of functioning, that is, body function and structure (condition or disorder), activity limitations, participation and contextual factors (environmental and personal factors). Disability is therefore viewed as a complex interplay between these three levels of functioning. In the ICF (WHO 2001) disability is therefore defined as an:

[*U*]mbrella term for impairments, activity limitations and participation restrictions that denotes the negative aspects of the interaction between a person’s health condition and their contextual factors i.e., environmental and personal factors. (p. 213)

In other words, the ICF does not attribute disability as a result of the impairment an individual presents with, but as an experience with the environment they function in. The ICF interrelates with the ecological-system approach which is used within vocational rehabilitation to specifically identify factors that hinder or facilitate the participation of persons with disabilities in employment (Erickson et al. 2014; Lindsay et al. 2015).

The ICF’s definition of disability has been highly praised, however, its relevance to LMICs critiqued, mainly because of the model’s view of the environment as disabling and not necessarily as a cause of disability (Visagie et al. 2017). In LMICs, there is a strong association between poverty, health and disability (Banks et al. 2017; Groce et al. 2011). For instance, the development of certain diseases can be because of lack of access or availability of health services (e.g. lack of access to medication, rehabilitation and assistive devices) and poor living conditions (e.g. malnutrition and poor water and sanitation) (Mitra et al. 2013). Therefore, diseases are a result of poverty caused by the environment. Nonetheless, the ICF is currently the most widely used comprehensive model of disability which is also adopted by the World Report on Disability (WHO & World Bank 2011). This study follows the definition of disability as used in the ICF. It should be noted that inconsistent definitions of disability were used in the studies included in the review.

The paucity of research on disability and employment in LMICs necessitated a scoping review. This allowed for the collation of existing literature to highlight existing gaps in research.

## Methods

The review followed the methodology for scoping reviews as outlined by Tricco et al. (2018). It aimed to specifically determine existing barriers and facilitators to the employment of persons with disabilities in LMICs. The review was guided by the following research question, ‘what are the barriers to and facilitators of the employment of persons with disabilities in LMICs?’.

### Search strategy

A multi-faceted search strategy was utilised including a systematic search of multiple electronic databases spanning the interval from 2008 to April 2020, which included Africa Wide Information, CINAHL, EconLit, Education Resources Information Center (ERIC), Medical Literature Analysis and Retrieval System Online (MEDLINE) business source complete and PsychInfo to avoid database bias (Munn et al. 2018). Search-terms were determined according to the suitability of each electronic database. Furthermore, publications from the WHO, the World Bank, the United Nations, the International Labour Organisation and other organisations such as professional and organisational associations were explored. Also, a search on Google Scholar, and a broad search on a web search engine, Google^TM^ were conducted.

The search strategy included a combination of key PCC concepts including *disability* (population), *employment (concept)* and *LMICs* (context) as indicated by the World Bank country income classification system (2019–2020). Appendix Table 1-A1 provides information on the search strategy used in this study. Following the completion of the search strategy in April of 2020, relevant studies related to the employment of persons with disabilities in LMICs were included using the exclusion and inclusion criteria outlined in Table 1.

**TABLE 1:** Inclusion and exclusion criteria.

Category	Inclusion criteria	Exclusion criteria
Targeted population	Persons with disabilities with childhood and acquired disabilities. Female and male participants who are economically active, that is, individuals who were considered economically active and were therefore 15 years and older.	Individuals with a disability because of ageing, chronic medical conditions such as HIV/AIDS, stroke and dementia as well as psychiatric disabilities were excluded. Children with disabilities and people older than 60 years.
Study period	Published peer-reviewed research studies dated from 2008 to April 2020.	Non-peer-reviewed articles were excluded as well as peer-reviewed articles published before the year 2008.
Study design	Studies following quantitative, qualitative and mixed-method designs were included.	Policy reports, analysis, dissertations and book chapters, editorials, opinion pieces, scoping and systematic reviews were not considered.
Language	Only articles published in English were included.	Articles published in languages other than English were excluded.
Study outcome	Studies reporting on employment including recruitment, hiring and vocational training of persons with disabilities, customised employment and self-employment were included.	Studies reporting on psychiatric/mental and medical disabilities, as well as studies reporting on transition*ing* from school to work and return to work, were excluded.
Context	Studies conducted in LMICs as listed in the World Bank (2019–2020) income classification were included. Studies that compared data between HICs and LMICs were also considered, provided the data could be segregated.	Studies conducted in HICs.

LMIC, low- and middle-income countries; HIV/AIDS, human immunodeficiency virus/acquired immunodeficiency syndrome; HICs, high-income countries.

### Data analysis

A data extraction tool was developed to extract information on the scope of the article. The tool included population, type of disability, aims of the study, design, context and the outcomes of the studies. An example of how data were extracted using the tool is depicted in Table 2. The data extraction was conducted by REM and SD. To determine factors that were reported as barriers and facilitators, identified studies were transferred to a computer-aided qualitative data analysis program, Atlas-ti^TM^ software, where the findings of the included studies were thematically analysed and coded. The identified codes were organised according to the second-level category classification of the ICF using refined linking rules as outlined by Cieza et al. (2019). The findings were therefore presented under the domains of the ICF, that is, body function and structure, activity and participation, environmental and personal domain (Table 3). To ensure accurate analysis of data, 20% of the total coded data were randomly selected and analysed by the second author, SD. Disagreements in coding were resolved by the first and second authors re-coding the data together.

**TABLE 2:** Studies reporting on barriers and facilitators of employment of persons with disabilities in low- and middle-income countries.

Authors and year of publication	Aim of the study	Study design/methods	Participants	Low- and middle-income country
Agyei-Okyere et al. (2019)	To document the perceptions and experiences of persons with disabilities concerning farming activities.	Qualitative: Individual interviews and focus group discussions	Nineteen persons with disabilities	Ghana
Amin and Abdullah (2017)	To explore the employment experience of Malaysian women with physical impairment.	Qualitative: Individual interviews	Thirty three Malaysian women with physical disabilities	Malaysia
Bhanushali (2016)	To explore the socio-economic conditions of persons with disabilities who are self-employed.	Quantitative: Survey	Two hundred persons with hearing, speech and physical disabilities	India
Bengisu and Balta (2011)	To determine a collective expert view on key issues regarding the employment of the workforce with disabilities in the hospitality industry.	Delphi survey	Forty three participants in three groups Researchers and disability experts Career experts Managers	Turkey
Bengisu, Izbirak and Mackieh (2008)	To determine the physical, attitudinal and organisational barriers faced by persons who are visually impaired.	Quantitative: Survey	One hundred and forty four employed and 54 unemployed persons with visual disabilities	Turkey
Bualar (2014)	To investigate the barriers affecting the employment opportunities of rural women with physical disabilities.	Qualitative: Semi-structured interviews	Ten women with physical disabilities	Thailand
Coelho et al. (2013)	To explore the factors that restrictions in the workplace are experienced by persons with disabilities.	Qualitative: Semi-structured interviews and observations	Thirty employed persons with disabilities	Brazil
Harun et al. (2020)	To describe the employment experiences of persons with learning disabilities.	Quantitative: Survey	Ninety, young persons with learning disabilities	Malaysia
Cramm et al. (2013)	To compare barriers to employment amongst disabled and non-disabled youth.	Quantitative: Survey	Four hundred and sixty six youth with a disability and 523 youth without a disability	South Africa
Santos Rodrigues et al. (2013)	To explore the use of youth apprenticeships and customised employment to improve workforce outcomes amongst persons with disabilities.	Qualitative: Case study	Two persons with disabilities	Brazil
Gudlavalleti et al. (2014)	To explore the health needs and barriers to accessing health services by persons with disabilities.	Quantitative: Survey	Eight hundred and thirty nine persons with disabilities (physical, visual, hearing and intellectual disabilities) matched to 1153 persons without disabilities	India
Khoo, Tiun and Lee (2013)	To explore the experiences regarding employment from persons with physical disabilities.	Mixed method: Semi-structured interviews and surveys	Two hundred and eighty seven persons with physical disabilities	Malaysia
Maja et al. (2011)	To identify the knowledge, attitudes and experiences of employers when hiring persons with disabilities.	Qualitative: Individual interviews	Three managers and two companies	South Africa
Malle et al. (2015)	To investigate prevailing challenges and opportunities for the participation of students with disabilities in vocational education programs.	Mixed-method: Individual interviews, observations and surveys	Hundred and ten trainers, 28 students with disabilities, 30 administrators	Ethiopia
Marsay (2014)	To explore ways of facilitating gainful employment for persons with disabilities.	Qualitative: Individual interviews	Fourteen persons with physical, intellectual, medical, learning and sensory disabilities	South Africa
Lamichhane (2012)	To explore the life-changing experiences of persons with disabilities brought by employment.	Quantitative: Survey	Four hundred and twenty three persons with visual, hearing and physical disabilities	Nepal
Lee, Abdullah and Mey (2011)	To identify drivers and inhibitors of employment for persons with disabilities.	Qualitative: Structured interviews	Twenty four teachers with a visual disability	Malaysia
Naami, Hayashi and Liese (2012)	To describe the issues associated with the unemployment of women with physical disabilities in Tamale, Ghana.	Qualitative: Individual interviews, and focus group discussions	Twenty four women with physical disabilities, 14 disability stakeholders	Ghana
Ned and Lorenzo (2016)	To describe the capacity of service providers in facilitating the participation of disabled youth in economic development opportunities.	Qualitative: Individual interviews and focus group discussions	Four family members, six service providers.	South Africa
Opoku et al. (2017a)	To explore barriers to employment of persons with disabilities.	Qualitative: Semi structured interviews	Thirty persons with physical, hearing and visual disabilities	Kenya
Opoku et al. (2017b)	To examine from the perspectives of participants, the life experiences of persons with disabilities 7 years after the ratification of the CRPD.	Qualitative: Focus group discussions	Thirty six persons with sensory and physical disabilities	Cameroon
Potgieter, Coeertze and Ximba (2017)	To explore the perceptions of individuals living with a disability with regard to career advancement challenges they face in the workplace.	Qualitative: Semi-structured interviews	Fifteen employed persons with disabilities	South Africa
Saigal and Narayan (2014)	To identify various physical barriers limiting the accessibility of persons with disabilities in the formal sector.	Quantitative: Survey	Fifty employed persons with visual and physical disabilities	India
Ta, Wah and Leng (2011)	To investigate employers’ perspectives towards employing persons with disabilities and to identify factors that promote or hinder the gainful employment of persons with disabilities.	Quantitative: Survey	Thirty nine employers from private companies	Malaysia
Ta and Leng (2013)	To explore and understand the challenges that are encountered by Malaysians with disabilities in the world of employment.	Mixed-method: Survey, face-to-face interviews and focus group discussion	Four hundred and seventy eight persons with physical, intellectual and sensory disabilities, 39 employers	Malaysia
Toldrá and Santos (2013)	To identify facilitators and barriers faced by persons with disabilities in the workforce.	Qualitative: Semi-structured interviews	Ten employees with disabilities	Brazil
Wiggett-Barnard and Swartz (2012)	To identify facilitating factors for the entry of persons with disabilities into the labour market.	Quantitative: Survey	Eighty six human resource managers	South Africa
Wolffe, Ajuwon and Kelly (2013a)	To evaluate the work experiences of employed individuals with visual impairments.	Qualitative: Interviews	Hundred and seventy two employed blind or partially sighted adults	Nigeria
Wolffe, Ajuwon and Kelly (2013b)	To report on the status of individuals in Nigeria who are visually impaired and successfully employed.	Quantitative: Survey	Hundred and seventy two employed blind or partially sighted adults	Nigeria
Yazıcı, Şişman and Kocabaş (2011)	To determine disabled people’s problems in the world of work.	Quantitative: Two separate surveys	Thirty two companies; 31 employers; 421 persons with disabilities	Turkey
Yusof et al. (2014)	To identify the employability and working patterns of vocational school leavers with disabilities.	Quantitative: Survey	Ninety nine students with sensory and learning disabilities	Malaysia
Yusof, Ali and Salleh (2015)	To explore the views of employers who hired youth workers with disabilities.	Qualitative: Semi-structured interviews	Three employers	Malaysia

CRPD, Convention on the Rights of Persons with Disabilities.

**TABLE 3:** Identified factors within the International Classification of Functioning, Disability and Health framework domains.

Domains of the ICF	Number of studies (*n*)	Included studies
**Body function and body structure**		
Type and severity of disability	8	Amin and Abdullah (2017); Bengisu and Balta (2011); Bhanushali (2016); Lamichhane (2012); Maja et al. (2011); Ned and Lorenzo (2016); Wolffe et al. (2013b); Yazıcı et al. (2011)
Health condition	5	Bualar (2014); Coelho et al. (2013); Cramm et al. (2013); Gudlavalleti et al. (2014); Ta et al. (2011)
**Activity and participation**		
Schooling	8	Bhanushali (2016); Coelho et al. (2013); Cramm et al. (2013); Lee et al. (2011); Malle et al. (2015); Opoku et al. (2017a); Yazıcı et al. (2011); Yusof et al. (2014, 2015)
Work and employment	7	Agyei-Okyere et al. (2019); Amin and Abdullah (2017); Bhanushali (2016); Cramm et al. (2013); Harun et al. (2020); Khoo et al. (2013); Ta and Leng (2013)
**Environmental factors**		
Attitudes	20	Amin and Abdullah (2017); Bengisu et al. (2008); Bengisu and Balta (2011); Bualar (2014); Coelho et al. (2013); Cramm et al. (2013); Khoo et al. (2013); Lee et al. (2011); Maja et al. (2011); Malle et al. (2015); Marsay (2014); Naami et al. (2012); Ned and Lorenzo (2016); Opoku et al. (2017a, 2017b); Potgieter et al. (2017); Ta et al. (2011); Ta and Leng (2013); Toldrá and Santos (2013); Yazıcı et al. (2011)
Services and systems	14	Amin and Abdullah (2017); Bengisu et al. (2008); Bualar (2014); Coelho et al. (2013); Cramm et al. (2013); Gudlavalleti et al. (2014); Khoo et al. (2013); Malle et al. (2015); Marsay (2014); Naami et al. (2012); Ta and Leng (2013); Wiggett-Barnard and Swartz (2012); Wolffe et al. (2013a); Yazıcı et al. (2011)
Policy and legislation	10	Amin and Abdullah (2017); Harun et al. (2020); Lamichhane (2012); Lee et al. (2011); Malle et al. (2015); Marsay (2014); Saigal and Narayan (2014); Wiggett-Barnard and Swartz (2012); Wolffe et al. (2013a); Yazıcı et al. (2011)
Natural and built environment	9	Amin and Abdullah (2017); Bengisu et al. (2008); Bualar (2014); Lamichhane (2012); Saigal and Narayan (2014); Ta and Leng (2013); Toldrá and Santos (2013); Wiggett-Barnard and Swartz (2012); Yazıcı et al. (2011)
Products and technology	7	Agyei-Okyere et al. (2019); Bengisu et al. (2008); Coelho et al. (2013); Saigal and Narayan (2014); Wolffe et al. (2013a, 2013b); Yazıcı et al. (2011)
Support and relationships	7	Bengisu et al. (2008); Bualar (2014); Harun et al. (2020); Lee et al. (2011); Marsay (2014); Opoku et al. (2017a); Ta and Leng (2013)
**Personal factors**		
Educational qualifications and vocational skills	20	Amin and Abdullah (2017); Bengisu et al. (2008); Bengisu and Balta (2011); Bhanushali (2016); Bualar (2014); Coelho et al. (2013); Cramm et al. (2013); Khoo et al. (2013); Lamichhane (2012); Lee et al. (2011); Maja et al. (2011); Naami et al. (2012); Opoku et al. (2017a, 2017b); Ta et al. (2011); Ta and Leng (2013); Toldrá and Santos (2013); Wolffe et al. (2013a, 2013b); Yazıcı et al. (2011)
Gender and age	11	Bengisu and Balta (2011); Bhanushali (2016); Bualar (2014); Coelho et al. (2013); Gudlavalleti et al. (2014); Harun et al. (2020); Naami et al. (2012); Ta and Leng (2013); Wolffe et al. (2013a, 2013b); Yazıcı et al. (2011)
Disability onset	3	Coelho et al. (2013); Wolffe et al. (2013a, 2013b)
Marital status	3	Bengisu et al. (2008); Wolffe et al. (2013a); Yazıcı et al. (2011)

ICF, International Classification of Functioning, Disability and Health framework.

### Ethical considerations

This article followed all ethical standards for research without direct contact with human or animal subjects.

## Results

**FIGURE 1:**
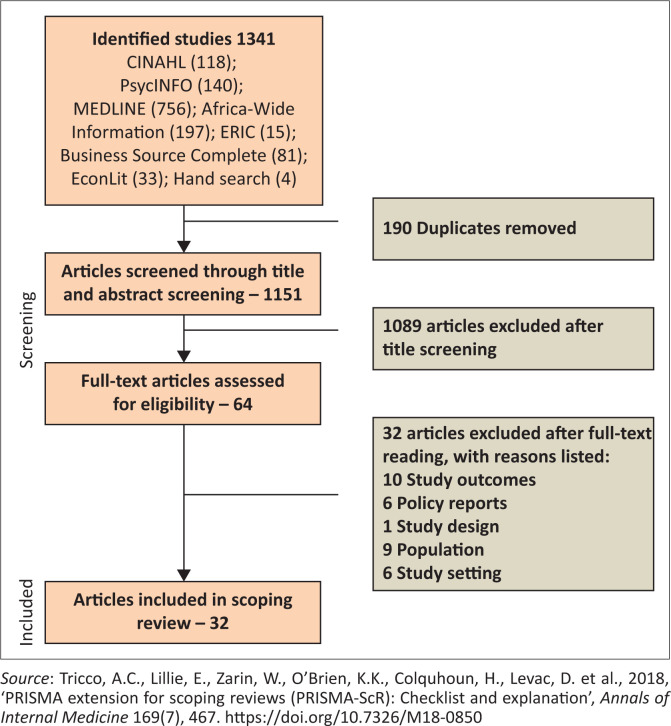
Preferred reporting items for systematic reviews and meta-analyses extension for scoping reviews.

An initial search was conducted in June 2019 which included studies between the years 1997 and 2019. This electronic search of the literature yielded a total of 1490 potentially relevant, peer-reviewed studies. When updating the review search strategy in April 2020, the authors made a decision to include studies dated between 2008 and 2020; this was done with the intention to only identify studies published after the ratification of the CRPD (United Nations 2006) by most LMICs. The final search strategy yielded a total of 1337 studies. The identified studies were then exported to Covidence^TM^, a web-based software platform that organises reviews such as systematic reviews (Babineau 2014). Following the exclusion of duplicates, a total of 1151 studies were independently screened by R.E.M. and S.D. at a title level. Finally, following the screening at an abstract level, 64 studies were assessed for eligibility, 24 of which met the inclusion criteria. Eight studies identified through hand searches and a search on Google^TM^ were added to the 24 studies which totalled to 32 included studies. Where there were conflicts, the authors reviewed the articles together and came to a consensus. Preferred reporting items for systematic reviews and meta-analyses extension for scoping reviews (PRISMA-ScR) (Tricco et al. 2018) were used to report on the scoping review process. Further information regarding the review process is charted in Figure 1.

Thirty-two studies were included in the final analysis as shown in Table 2. Geographical distribution of the countries represented in the review as classified by the World Bank classification (2019–2020) included two studies from low-income countries (6.3%), nine from lower-middle-income countries (28%) and 21 from upper-middle-income countries (66%). Countries represented in the review included Malaysia (*n* = 8), South Africa (*n* = 6), India (*n* = 3), Brazil (*n* = 3), Turkey (*n* = 3), Nigeria (*n* = 2), Ghana (*n* = 2), whilst the rest of the studies were from Cameroon, Ethiopia, Kenya, Nepal and Thailand. Sixteen qualitative (50%), 13 quantitative (41%) and three mixed-method (9%) original studies were included.

The included studies mainly focused on exploring the experiences of persons with disabilities and views of employers with regard to economic participation (31; 97%), whilst two specifically focused on vocational training (Malle, Pirttimaa & Saloviita 2015; Yusof, Ali & Salleh 2014) and one on integrative employment (Santos Rodrigues et al. 2013). Although the included studies covered various types of disabilities such as sensory, intellectual, physical, learning, communication and multiple disabilities (Table 2), there was a vast representation of sensory disabilities, particularly visual disabilities (8; 24%).

The participants in the studies varied, 24 studies explored the experiences of persons with disabilities themselves (27; 75%), whilst seven studies explored the views of employers (22%), and three studies explored the perspectives of family members, recruitment agencies and other stakeholders (researchers and educators). Although studies included both male and female participants, three studies focused specifically on women with disabilities (Amin & Abdullah 2017; Bualar 2014; Naami, Hayashi & Liese 2012). Table 1 provides a list of studies reporting on barriers and facilitators of the employment of persons with disabilities in LMICs.

### Barriers and facilitators identified within the International Classification of Functioning, Disability and Health framework domains

Table 3 provides a summary of factors reported to either hinder or facilitate the employment of persons with disabilities as described in the studies included in the review.

The study used the ICF as a guiding framework; therefore, the identified barriers and facilitators are reported according to its domains, that is, body function and body structure, activity and participation, environment and personal domain. The vast majority of studies (32; 97%) were reported on barriers to employment, whilst only nine studies (27%) were reported on facilitators of employment. With regard to the ICF, included studies reported on factors related to multiple domains of the ICF (Table 1), with only four studies (12%) reporting on factors within one domain. An example would be a study by Saigal and Narayan (2014) that reported on inaccessible environments as a barrier to employment, which solely lies within the environment domain.

Barriers are reported in the study as a ‘lack of’ and facilitators as ‘availability of’. It should be noted, however, that a lack of a barrier is not automatically seen as a facilitator, although the absence or lack of a facilitating factor can be a barrier. Identified factors that are barriers and facilitators are, therefore, reported together.

Thirteen studies (39%) reported on factors within the body function and body structure domain which included the type and severity of disability (8; 62%), and health condition (5; 38%). Fifteen studies (47%) were reported on factors within the activity and participation domain, including admission to schooling (8; 53%) and work and employment (7; 47%). Twenty-two (69%) studies were reported on personal factors, namely educational qualifications and vocational skills (20; 91%), gender and age (11; 50%), and three studies were reported on the onset of the disability and marital status. Most of the studies were reported on factors within the environment (28; 88%). The presence of attitudes was reported as a major contributing factor to the unemployment of persons with disabilities (20; 71%) whilst other factors were linked to services and systems (14; 50%), policy and legislation (10; 36%), natural and built environment (9; 32%), products and technology (7; 25%) and support and relationships (7; 25%).

## Discussion

This study aimed to explore existing literature on barriers and facilitators to the employment of persons with disabilities in LMICs. The results of the review were aligned to the domains of the ICF. Similar to previous reviews, results indicated a paucity of research regarding the economic participation of persons with disabilities in LMICs (Ebuenyi et al. 2018; Tripney et al. 2019). As the included studies were published post the ratification of the United Nations Convention on the Rights of Persons with Disabilities (UN CRPD) (United Nations 2008) and its optional protocols by the majority of the LMICs, it was therefore assumed that most countries had initiatives in place aimed at eradicating and promoting equal rights. However, despite these efforts, the included studies further reiterate the paucity of research in LMICs with regard to the employment of persons with disabilities and secondly, the poor advancement in the participation of persons with disabilities in the open labour market. Furthermore, the included studies do not, unfortunately, represent half of the listed LMICs, and only 12 (22%) out of 54 countries were represented in the review.

The study used the ICF as a guiding framework. This enabled an in-depth understanding of challenges and facilitators within the microsystem (i.e. individual-level), mesosystem (i.e. immediate environment) and the macro-system (i.e. societal level). Barriers and facilitators identified were mainly reported in the environment (27; 87%) and personal (23; 74%) domain. Similar to previous studies, 90% of the studies in the review mainly reported on hindering factors as opposed to facilitating factors to the employment of persons with disabilities. This could be attributed to the need to first establish and understand existing barriers to employment of persons with disabilities in LMICs prior to solutions being sought (Ebuenyi et al. 2018).

The reported findings have some commonality to those reported in HICs (Hästbacka et al. 2016; Khayatzadeh-Mahani et al. 2019; Padkapayeva et al. 2017; Vornholt et al. 2018), however, as observed by Mitra and Sambamoorthi (2014), HICs report more on activity limitation, whilst LMICs mostly report on limitations imposed by the disability, therefore an individual is perceived disabled on the virtue of the presence of impairment regardless of whether or not they experience restrictions to participation in daily life situations.

### Body function and body structure

The severity and type of disability determine the likelihood of one being employed and also the willingness of employers in hiring a person with a disability (Amin & Abdullah 2017; Bengisu & Balta 2011; Maja et al. 2011). In Amin and Abdullah’s (2017) study, employers rejected persons with physical disabilities, citing inaccessible workspaces as the reason for the rejection. Similarly, in a study by Maja et al. (2011), organisations interviewed and reported that the working environments in their companies were not suitable for persons with physical disabilities as a high level of movement and endurance was required. Also, certain job descriptions were reported as not suitable for certain types of disabilities (Ned & Lorenzo 2016), for example, persons with visual and physical disabilities were limited in terms of variety of job positions (Bengisu & Balta 2011; Lamichhane 2012). Visual disabilities were represented in most studies in the review, perhaps highlighting that this population is more likely to be employed in LMICs. Lamichhane (2012) found an explanation of this phenomenon, wherein 43.42% of persons with visual disabilities in his study were employed within the education profession. This was as a result of advocacy movements in the 1980s that called for the inclusion of persons with disabilities in education colleges and thereby demanded that the government provide support in terms of assistive technology and adapted material.

In the literature, persons with severe disabilities are reported to be disadvantaged in terms of employment opportunities available in LMICs (Mizunoya & Mitra 2013). Likewise, the studies in the review reported the lack of employment opportunities available for persons with disabilities. In a study by Yazici et al. (2011), employers showed a preference in hiring individuals whose disability was less severe in nature, that is, presented with 100% hearing, vision and communication skills (Yazıcı et al. 2011). In Bhanushali (2016), 92% of the participants whose disability was severe in nature opted for self-employment because of the barriers experienced with securing employment. From the findings, it can be deduced that the lack of employment opportunities paints a bleak future outcome. Given the lack of employment opportunities in LMICs, the option of self-employment/entrepreneurship should be further explored for persons with disabilities particularly those who present with a severe disability.

Another hindering factor, poor health was reported to also negatively impact employment outcomes, as frequent sick-leave is required which means time away from work (Bualar 2014). Cramm et al. (2013) found that the unemployment of the majority of the 523 youth with disabilities was associated with poor health. Equally, Gudlavalleti et al. (2014) found that 18.4% of 839 persons with disabilities who participated in the study required medical services more often than those without a disability. It is known that many persons with disabilities have co-morbid or secondary conditions in addition to their disability, and therefore require greater medical attention than their counterparts without a disability (Bright, Wallace & Kuper 2018). It should be noted that poor health in persons with disabilities in LMICs is linked to a lack of access and the unavailability of rehabilitative services and medical care (Lorenzo & Cramm 2012; Mitra et al. 2013). The findings, therefore, highlight the fact that the participation in the employment of persons with disabilities in LMICs can be enhanced by ensuring access to medical and rehabilitative services as part of intervention programmes (Abdel Malek, Rosenbaum & Gorter 2020; Cawood & Visagie 2015).

### Activity and participation

Persons with disabilities encounter barriers to participation in major life activities such as education and employment. In this review, the most frequently mentioned barrier to participation in major life areas was the lack of access to schooling (i.e. the lack of access to basic, higher education and vocational training) (Bhanushali 2016; Cramm et al. 2013; Yazıcı et al. 2011; Yusof et al. 2014). This impacts the acquisition of job-related skills that are required for one to be employed (Cramm et al. 2013; Lee et al. 2011). Malle et al. (2015) reported that barriers experienced by persons with disabilities from participating in vocational education were because of the lack of adapted curriculum and educational material, skilled educators and trainers, as well as systemic exclusion from certain types of courses. Also, Yusof et al. (2014) found that persons with disabilities who had graduated from a vocational training programme were employed in positions not related to their qualifications, many of which were in low-paying positions. These results highlight the poor link between skills required in the field and skills provided in vocational training programmes. It is therefore imperative to have an alignment in the type of skills training provided and skills that are in demand in the open labour market (Opini 2010).

Again as reported by studies in the review, employment opportunities were scarce for persons with disabilities (Harun et al. 2020; Khoo et al. 2013; Ta & Leng 2013). Where opportunities were available, they were in low-paying positions that required low-level skills (Amin & Abdullah 2017; Agyei-Okyere et al. 2019; Bhanushali 2016). In a study by Khoo et al. (2013), participants with physical disabilities reported unequal employment opportunities, and the government prioritises employment of the skilled able-bodied population (Khoo et al. 2013). Notably, the focus in most studies in the review was specific to the formal sector, with work based in urban areas (Potgieter et al. 2017; Saigal & Narayan 2014; Wiggett-Barnard & Swartz 2012; Wolffe et al. 2013a). Given that most LMICs rely on self-employment (Mitra et al. 2013), the informal sector was scarcely mentioned (Agyei-Okyere et al. 2019; Bhanushali 2016). For those deciding to start businesses, support in the form of funding from governments is poor (Agyei-Okyere et al. 2019; Bhanushali 2016). Agyei-Okyere et al. (2019) indicated barriers that persons with disabilities faced in participating in the farming business, which were related to a lack of financial support from bank institutions and the government. Similarly, studies in the literature also reiterate that vocational training programmes in LMICs should focus on skills related to the development of businesses and understanding models of funding to sustain those businesses (Tripney et al. 2019).

Integrative employment was a reported facilitator to employment for persons with severe disabilities (Amin & Abdullah 2017; Santos Rodrigues et al. 2013). According to Santos Rodrigues et al. (2013), customised employment provides skills training opportunities, work preparation programmes, and integrates persons with disabilities in employment by linking them to potential employers and business opportunities. In a study by Amin and Abdullah (2017), supported employment workshops that provided employment opportunities to women with physical disabilities were located in remote areas far from urban areas where social and economic activities occur, not to mention that work in these workshops was not only non-stimulating but was of minimal wage. Similar findings are reported in the literature, where the benefits of integrative employment programmes, such as customised and supported employment programmes, are highlighted in the literature, and these programmes facilitate the integration of this population into the open labour market (Tinta, Steyn & Vermaas 2020). The programmes are further said to provide an opportunity for the development of skills required for gainful employment whilst accommodating the needs of persons with severe disabilities (García-Villamisar, Wehman & Diaz Navarro 2002).

### Environmental factors

Previous studies have identified barriers and facilitators to be mainly within the environment (Hästbacka et al. 2016; Khayatzadeh-Mahani et al. 2019; Lindsay 2011). In this review, factors were identified within all chapters of the environmental domain, again highlighting the influence of the environment on functioning (Glässel et al. 2011). The most frequently reported factors in this review were attitudes, policies and legislation as well as services and systems.

Negative attitudes from employers, family and society were reported as major factors that hinder participation in employment. Employers’ misconceptions held about disability influence hiring practices (Bengisu et al. 2008; Bualar 2014; Potgieter et al. 2017). Employers lack trust and believe that persons with disabilities can be as productive as other employees without disabilities (Lee et al. 2011; Maja et al. 2011; Toldrá & Santos 2013). Furthermore, in a study by Ta et al. (2011), employers reported a lack of knowledge in managing persons with disabilities in the workplace. Persons with disabilities are often perceived by families as incapable of being educated and employed (Khoo et al. 2013; Naami et al. 2012). In extreme cases, persons with disabilities face abandonment from their families as a result of their disability (Bualar 2014; Harun et al. 2020; Ta & Leng 2013). In the same light, support from family is a notable facilitator (Bengisu et al. 2008; Opoku et al. 2017a). Marsay (2014) found that 40% of the interviewed participants with disabilities who were employed reported that support from family and friends played a crucial role in their staying in their job.

The lack of education services (i.e. inclusive and well-resourced schools facilitate the acquisition of skills crucial for employment) (Malle et al. 2015; Naami et al. 2012; Ta & Leng 2013), transportation (Amin & Abdullah 2017; Bualar 2014; Khoo et al. 2013) and health services (Bengisu et al. 2008; Coelho et al. 2013; Cramm et al. 2013) hinders participation in employment. A systematic review conducted on the barriers to accessing rehabilitative services in LMICs indicated that 22 of the 77 included studies were related to distance and transportation challenges, affordability of services, fear and lack of knowledge about the importance of services (Bright et al. 2018). Other services such as employment services (Bengisu et al. 2008; Cramm et al. 2013; Gudlavalleti et al. 2014; Wiggett-Barnard & Swartz 2012) and communication services (i.e. media such as radio, television and newspapers) (Amin & Abdullah 2017; Lee et al. 2011; Opoku et al. 2017a) were reported as facilitators to participation.

Also, the studies discussed the importance of the availability of legislation and policy that promote the participation of persons with disabilities in education and employment (Amin & Abdullah 2017; Harun et al. 2020; Lamichhane 2012). Yazici et al. (2011) found that 49.9% of the employees with a disability were employed by the Turkish Labour Institution as a result of the set government quota of 3%. Unfortunately, in LMICs, support from the government is limited, with the implementation of policies being poor. Implementation and enforcement of anti-discriminatory law and policies that facilitate the employment of persons with disabilities are therefore imperative.

### Personal factors

Facilitators to employment reported include interpersonal skills that facilitate employment such as academic (e.g. reading and writing), and job-related skills (Coelho et al. 2013; Harun et al. 2020; Lee et al. 2011; Yusof et al. 2015). Similarly, the lack of education limits employment opportunities available to an individual with a disability (Opoku et al. 2017a; Toldrá & Santos 2013). Khoo et al. (2013) found that 158 out of 287 persons with a physical disability (55%) encountered barriers to securing employment because of low levels of education. Important to realise, however, is the fact that the lack of access to education and the unavailability of education services and systems greatly contribute to poor levels of education (Mitra et al. 2013). These findings highlight the complex interplay between an individual’s condition and factors within the environment that either hinder or facilitate participation in employment.

Existing systems tend to favour men rather than women with men having increased access to education and employment opportunities (Amin & Abdullah 2017; Lamichhane 2012; Toldrá & Santos 2013). Naami et al. (2012) highlighted the double prejudice faced by women with disabilities in Ghana, firstly based on their gender and secondly on their disability. These prejudices are further complicated by issues of culture, religion, class and geographic location (Bualar 2014; Opoku et al. 2017a; Ta et al. 2011). Marital status increases the likelihood of being employed (Bengisu et al. 2008; Yazıcı et al. 2011). In a study by Wolffe et al. (2013b), persons with visual disabilities who were married worked more hours, experienced less difficulty in accessing learning and employment opportunities and earned more than those who were unmarried. Using the ICF, the multitude of factors that impact women with disabilities beyond their diagnosis could be identified. Persons with developmental disabilities were more likely to be found in employment than those with disabilities acquired later in life (Coelho et al. 2013; Wolffe et al. 2013a, 2013b). In the same light, age predicted whether one would be employed or not (Coelho et al. 2013; Wolffe et al. 2013a, 2013b). Older persons with disabilities were found to be in employment compared to those who were younger as they were found to be still pursuing some sort of educational qualification (Wolffe et al. 2013a).

Although the personal domain is not coded within the ICF, these results reiterate the influence of personal factors on functioning and subsequent participation in employment (Glässel et al. 2011). Intervention programmes should take into consideration an individual’s personal factors in addition to their diagnosis and identified factors within the environment (Momsen et al. 2019).

### Limitations of the study

A few limitations exist in this study. Firstly, only peer-reviewed journal articles and original studies were included in the review. The authors acknowledge that the inclusion of other sources such as dissertations and disability reports could have yielded a higher number of studies and therefore, richer information. Secondly, only studies published in English were included. However, English is not an official language in most LMICs. Future studies should thus consider the inclusion of studies in other common languages other than English. Lastly, a handful of LMICs were represented in the study and therefore results cannot be generalised. It is thus recommended that future studies include a wide representation of LMICs.

## Conclusion

The findings of this study ICF highlight the fact that persons with disabilities in LMICs still face marginalisation in participating in employment. The ICF proved to be a suitable tool for describing factors in LMICs that hindered and facilitated participation. In the review, contextual factors (personal and environmental factors) were found to be major barriers or facilitators to employment. This information indicates the influence of individual factors in addition to external factors on functioning. The findings should be taken into consideration by researchers, clinicians and policy makers when developing strategies aimed at increasing the participation of persons with disabilities in LMICs. Based on the findings from the study, it is recommended that future studies explore how the identified facilitators to employment of persons with disabilities can be practically implemented in LMICs.

## Appendix 1

**TABLE 1-A1:** Search strategy used in the study.

Criteria	Component	Terms
Population	Persons with disabilities	Disab*OR Condition OR Disorder OR Ailment OR Illness OR Malady OR Disease OR Disable OR Incapacity OR Special Need OR Handicap OR Abnormality OR Defect OR Impairment OR Developmental Delay OR Long-Term Health Conditions OR Childhood disability OR Restriction AND
Context	Low- and middle-income country	Countr* OR emerging econom* OR Developing Countr* OR Low middle income Countr* OR Low Income Countr* OR Middle Income Countr* OR Third World OR Underdeveloped Countr* OR Afghanistan OR Benin OR Burkina Faso OR Burundi OR Central African Republic OR Chad OR Comoros OR Congo OR Eritrea OR Ethiopia OR Gambia OR Guinea OR Guinea-Bissau OR Haiti OR Korea OR Liberia OR Madagascar OR Malawi OR Mali OR Mozambique OR Nepal OR Niger OR Rwanda OR Senegal OR Sierra Leone OR Somalia OR South Sudan OR Tanzania OR Togo OR Uganda OR Zimbabwe OR Armenia OR Bangladesh OR Bhutan OR Bolivia OR Cabo Verde OR Cambodia OR Cameroon OR Congo OR Côte d’Ivoire OR Djibouti OR Egypt OR El Salvador OR Ghana OR Guatemala OR Honduras OR India OR Indonesia OR Kenya OR Kiribati OR Kosovo OR Kyrgyz Republic OR Lao PDR OR Lesotho OR Mauritania OR Micronesia OR Moldova OR Mongolia OR Morocco OR Myanmar OR Nicaragua OR Nigeria OR Pakistan OR Papua New Guinea OR Philippines OR Samoa OR São Tomé And Principe OR Solomon Islands OR Sri Lanka OR Sudan OR Swaziland OR Syrian Arab Republic OR Tajikistan OR Timor-Leste OR Tonga OR Tunisia OR Ukraine OR Uzbekistan OR Vanuatu OR Vietnam OR West Bank And Gaza OR Yemen OR Zambia OR Albania OR Algeria OR American Samoa OR Angola OR Argentina OR Azerbaijan OR Belarus OR Belize OR Bosnia And Herzegovina OR Botswana OR Brazil OR Bulgaria OR China OR Colombia OR Costa Rica OR Cuba OR Dominica OR Dominican Republic OR Ecuador OR Equatorial Guinea OR Fiji OR Gabon OR Georgia OR Grenada OR Guyana OR Iran OR Iraq OR Jamaica OR Jordan OR Kazakhstan OR Lebanon OR Libya OR Macedonia OR Malaysia OR Maldives OR Marshall Islands OR Mauritius OR Mexico OR Montenegro OR Namibia OR Palau OR Panama OR Paraguay OR Peru OR Romania OR Russian Federation OR Serbia OR South Africa OR St Lucia OR St Vincent And The Grenadines OR Suriname OR Thailand OR Turkey OR Turkmenistan OR Tuvalu OR Venezuela AND
Concept	Employment	Employ* OR Trade OR Recruit* OR Income OR Hiring OR Work OR Job OR Vocation OR Business OR Entrepren* OR Workplace OR Occupation

Disab., disability/disabilities; Countr., country/countries; Entrepren., entrepreneur/entrepreneurship; Employ., employment; econom., economy/economies.
